# Supplementary material for: Chromatin environment-dependent effects of DOT1L on gene expression in male germ cells
Source: Commun Biol. 2025 Jan 28;8:138. doi: 10.1038/s42003-024-07393-x (PMC11775102; doi:10.1038/s42003-024-07393-x)

SUPPLEMENTARY MATERIAL

Chromatin Environment-Dependent Effects of DOT1L on Gene Expression in Male Germ Cells

Manon Coulée[1], Alberto de la Iglesia[1], Mélina Blanco[1,2,3], Clara Gobé[1], Clémentine Lapoujade[2,3], Côme Ialy-Radio[1], Lucia Alvarez-Gonzalez[4,5], Guillaume Meurice[6], Aurora Ruiz-Herrera[4,5], Pierre Fouchet[2,3], Julie Cocquet[1*], Laïla El Khattabi[1,7*]

[1] Université Paris Cité, CNRS, Inserm, Institut Cochin, F-75014 Paris, France.

[2]Université Paris Cité, CEA, Stabilité Génétique Cellules Souches et Radiations, Fontenay-aux-Roses, France.

[3] Université Paris-Saclay, CEA, Stabilité Génétique Cellules Souches et Radiations, Laboratoire des Cellules Souches Germinales, Fontenay-aux-Roses, France.

[4] Departament de Biologia Cel·lular, Fisiologia i Immunologia, Universitat Autònoma de Barcelona, Cerdanyola del Vallès, Spain

[5] Genome Integrity and Instability Group, Institut de Biotecnologia i Biomedicina, Universitat Autònoma de Barcelona, Cerdanyola del Vallès, Spain

[6]MOABI, plateforme de Bioinformatique de l'APHP, Paris, France

[7]Sorbonne Université, APHP Hôpital Pitié-Salpêtrière, Paris Brain Institute – ICM, INSERM U1127, CNRS UMR 7225, Paris, France

*These authors contributed equally to this work.

Correspondence to julie.cocquet@inserm.fr and laila.el-khattabi@aphp.fr

Supplementary Figure legends

Supplementary Figure 1. ChIP-Seq and CUT&Tag information.

a. Spearman correlation of H3K79me2 ChIP-seq data. **b**. Overall results from H3K79me2 ChIP-seq data. **c**. Spearman correlation of H3K27ac and H3K27me3 CUT&Tag data in RS.

Supplementary Figure 2. Dynamic of chromatin states associated to H3K79me2.

Chromatin states categories are defined by ChromHMM model (**Fig 2a**) **a.** Dynamic of bivalent chromatin states found in RS (TssBiv and EnhBiv). **b.** Dynamic of active chromatin states found in RS (TSSA, Tx, EnhG and EnhA). **c.** Dynamic of heterochromatin states during spermatogenesis (ZNF/Rpts and Het). **d.** Dynamic of weak chromatin states found in RS (TSSFlnk, TxWk and EnhW). **e.** Dynamic of quiescent chromatin states found in RS. For each figures, only dynamic with more than 100 regions are shown.

Supplementary Figure 3. **Correlation between gene expression and enhancer**.

Mean gene expression level (log(CPM)) of genes associated with enhancers enriched (+) or not (-) in H3K79me2 in GSC, SCI and RS. Top panels: method from ChromENVEE, and bottom panels: method from Rada-Iglesias et al.[87]. Stars indicate a p-value < 0.05 calculated using Wilcoxon test. Bivalent enhancers correspond to only 23 H3K79me2+ regions in GSC. The statistical analysis is therefore not powerful enough to confidently determine a p-value, which may explain the discrepancy between the two methods.

Supplementary Figure 4. *Dot1l*-KO. a. Western blot detection of H3K79me2 in GSCs from CTL and *Dot1l*-KO mice. The equivalent of 32µg of protein was loaded in each well. Anti-TUBULIN was used as a loading control. **b.** Western blot detection of H3K79me2 in purified male germ cells from CTL and *Dot1l*-KO mouse. The equivalent of 20µg of protein was loaded in each well. Anti-TUBULIN was used as a loading control. **c.** Expression level of marker genes in the 5 control male germ cell types and in Sertoli (the predominant somatic cell type in the testis). Values represent Log2 rpkm values normalized to the median. NB. *Spata16* is expected to be expressed from SCI onwards. **d**. Barplot representing the average length of deregulated genes (up, down) and not deregulated genes (spike-in RNA-Seq analysis). Gene size is divided into

percentile groups in each cell type. A star indicates a pvalue<0.05 found by comparison with not deregulated genes (using a Chi2 test). **e**. Functional annotation of deregulated genes (up, down) and not deregulated genes (spike-in RNA-Seq analysis). IG-TR: Immunoglobulin/T-receptor; lincRNA: long intergenic non-coding RNA; lnc: long non-coding RNA; miRNA: microRNA; misc_RNA: miscellaneous RNA (that are not in any other classification); mtRNA: mitochondrial RNA; Protein coding; Pseudogene; rRNA: ribosomal RNA; snoRNA: small nucleolar RNA; snRNA: small nuclear RNA; TEC: transcripts to be experimentally confirmed.

Supplementary Figure 5. RNA-Seq.

a. Volcano plots showing differentially expressed genes between *Dot1l*-KO *vs* CTL cells. **b**. GSEA for Kit- and Kit+ cells compared to results obtained for SCI, SCII and RS, which were previously shown in Blanco et al.[18] . **c.** MD plot showing deregulated genes in SCI and SCII following spike-in normalization (NB. MD plots without ERCC normalization were already shown in Blanco et al.[18]. Significantly downregulated genes that are enriched in H3K79me2 and known transcriptional repressors are indicated in the figure.

Supplementary Figure 6. Sex chromosome gene regulation.

a. Chromosome localization of differentially expressed genes (spike-in RNA-Seq analysis) in Kit-, Kit+ and SCI. Blue: downregulated genes (DR), red: upregulated genes (UR), the green dot indicates the centromere, and the grey dot, the telomere. **b**. Gene expression difference between *Dot1l*-KO and control, at different spermatogenesis cell stages according to the type of chromosomes. Boxplots show Log2-fold changes of *Dot1l*-KO relative to CTL gene expression according to the chromosome location, in each cell type, for all genes detected in the RNA-seq analyses (including not significantly deregulated). Box: 25th/75th percentiles. Bar in the box: median. Whiskers: 1.5 times the interquartile range from the 25th/75th percentiles. Dashed lines: log2(1.5) fold change. Stars indicate a significant FDR calculated using Wilcoxon test adjusted with Benjamini-Hochberg correction (*, p <0.05; ****, p <0.00005; ns, not significant). **c**. Quantification of transcript level by RT-qPCR in RS (n= 3 replicates for CTL and 5 for *Dot1l*-KO samples). Values represent the fold change relative to the control, normalized to the geometric mean of housekeeping genes, with the mean shown as a dash (the star indicates a p <0.05 obtained with a t-test adjusted for multiple tests). **d**. Ratio of peak numbers at RS stage compared to SCI stage, per chromosome, for several histone marks. The dashed line

indicates a ratio = 1 corresponding to a stable number of peaks. **e**. Ratio of peak numbers on the X or Y chromosome in RS vs SCI (circle) and in SCI vs GSC (triangle), for several histone marks. The dashed line indicates a ratio = 1

Supplementary Figure 7. ChIP-seq analyses. a. Peaks distribution of different histone marks along the chromosomes at three cell stages of spermatogenesis (GSC, SCI and RS). The number of peaks is normalized to the chromosome length. **b**. H3K27ac and H3K27me3 enrichment profiles at X-linked genes or autosomal genes from spike-in CUT&Tag experiments performed on 3 *Dot1l*-KO (KO) and 3 control (CTL) RS samples.

Supplementary Figure 8.

a. H3K79me2 peaks distribution according to their size (in bp). The blue line corresponds to the 9th percentile of the H3K79me2 peak size in RS. The red line corresponds to the 9th percentile of the region enriched in H3K79me2 after GSC, SCI and RS peaks were merged. **b.** Percentage of coverage of chromatin state. In black, the major chromatin state and in red, the second major chromatin state. Dashed line corresponding to median in first and second major chromatin state (black and red).

Supplementary data

Supplementary Data 1. Dynamic of H3K79me2 peaks from GSC to RS.

Supplementary Data 2. DEG genes using spike-in (ERCC) normalization.

Supplementary Data 3. Correlation between DEG and H3K79me2.

Supplementary Data 4. Correlation between DEG and chromatin environment.

Supplementary Data 5. Primer sequences.

References

1. Rada-Iglesias, A. *et al.* A unique chromatin signature uncovers early developmental enhancers in humans. *Nature* **470**, 279–283 (2011).

2. Blanco, M. *et al.* DOT1L regulates chromatin reorganization and gene expression during sperm differentiation. *EMBO Rep* **24**, e56316 (2023).

Supplementary Figure 1. ChIP-Seq and CUT&Tag information.

a

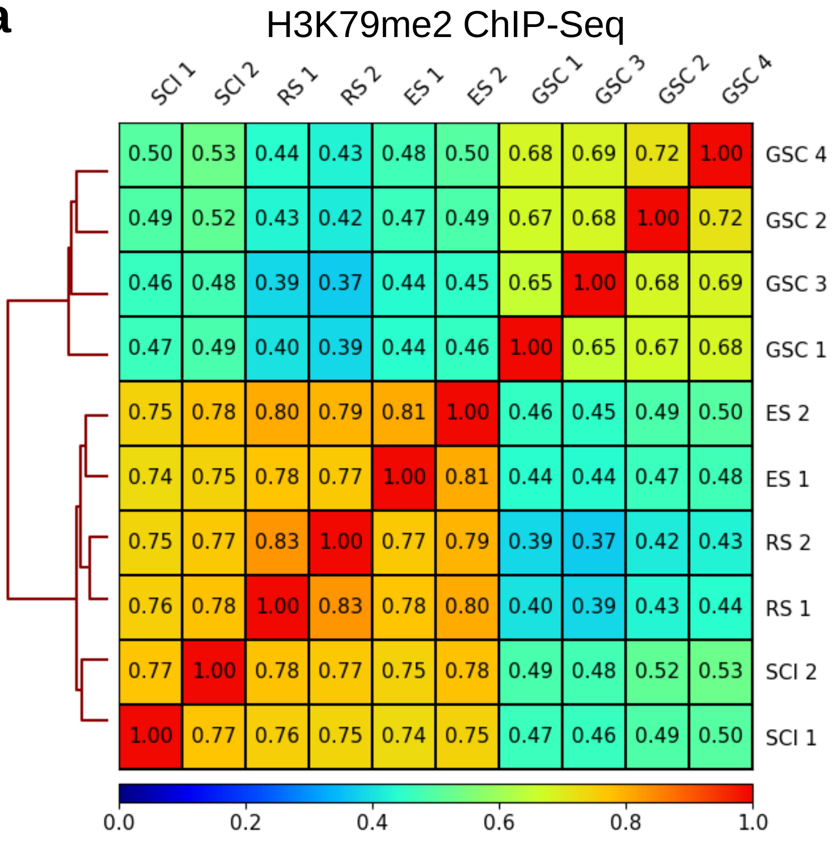

b

	GSC rep1	GSC rep2	GSC rep3	GSC rep4	SCI rep1	SCI rep2	RS rep1	RS rep2	ES rep1	ES rep2
Number of reads	21 260 743,00	23 051 274,00	24 537 676,00	25 195 852,00	45 210 180,00	54 045 728,00	56 859 200,00	53 051 834,00	43 379 619,00	55 919 709,00
**MACS2 peak calling (broad parameter)**										
Number of peaks	26 856,00	26 463,00	25 571,00	28 820,00	53 603,00	53 895,00	70 458,00	75 418,00	7 678,00	1 276,00
Number of annotated peaks	26 601,00	26 189,00	25 325,00	28 505,00	52 977,00	53 292,00	69 485,00	74 382,00	7 607,00	1 225,00
**common analysis**										
Number of peaks in common				15 896,00	46 034,00		67 918,00			385,00
**ChIPseeker**										
Number of peaks annotated as "promoter"	10 089,00				19 480,00		25 555,00		4 040,00	
Number of peaks annotated as "intragenic"	5 621,00				13 880,00		21 348,00		4 267,00	
Number of peaks annotated as "distal intergenic"				170,00	2 122,00		8 788,00			159,00
Number of peaks annotated as "downstream"				15,00		219,00		535,00		7,00

c

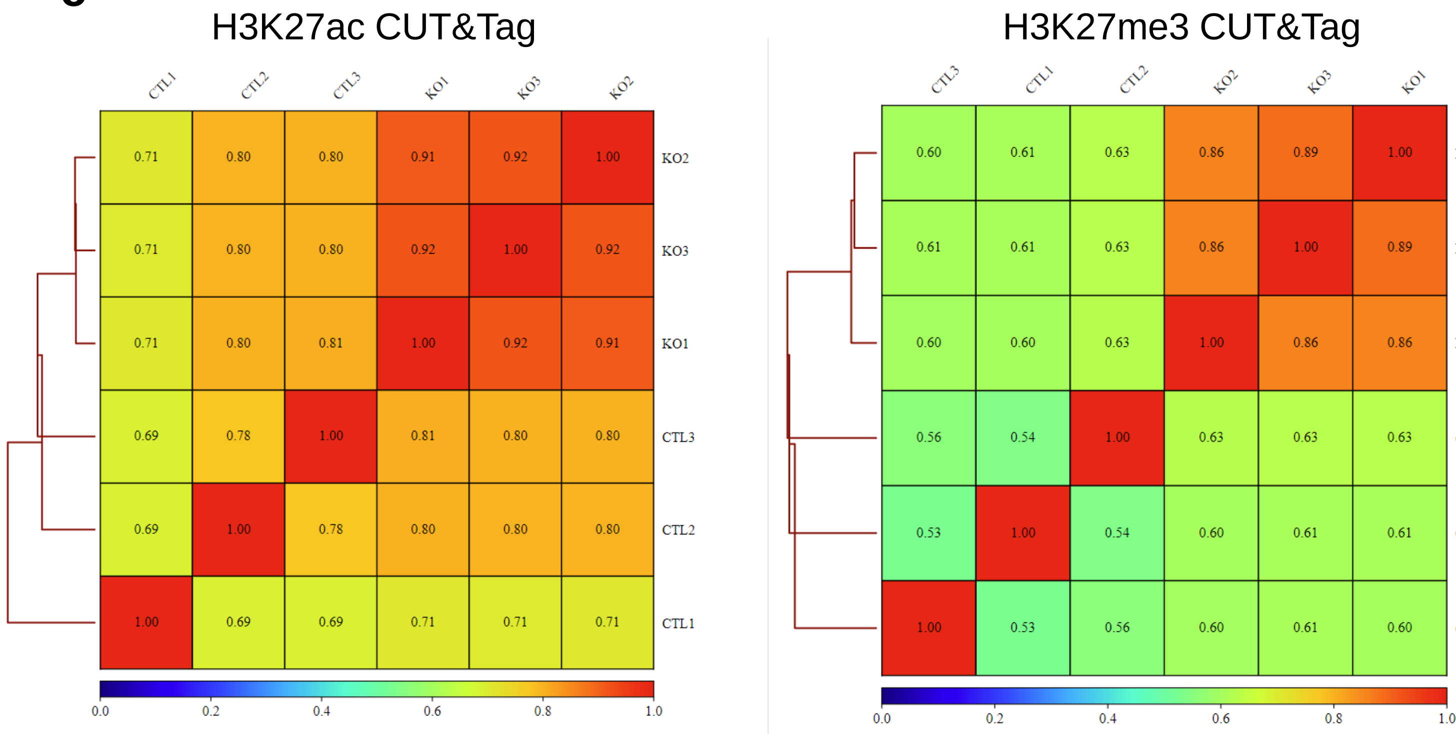

Supplementary Figure 2. Dynamic of chromatin states associated to H3K79me2.

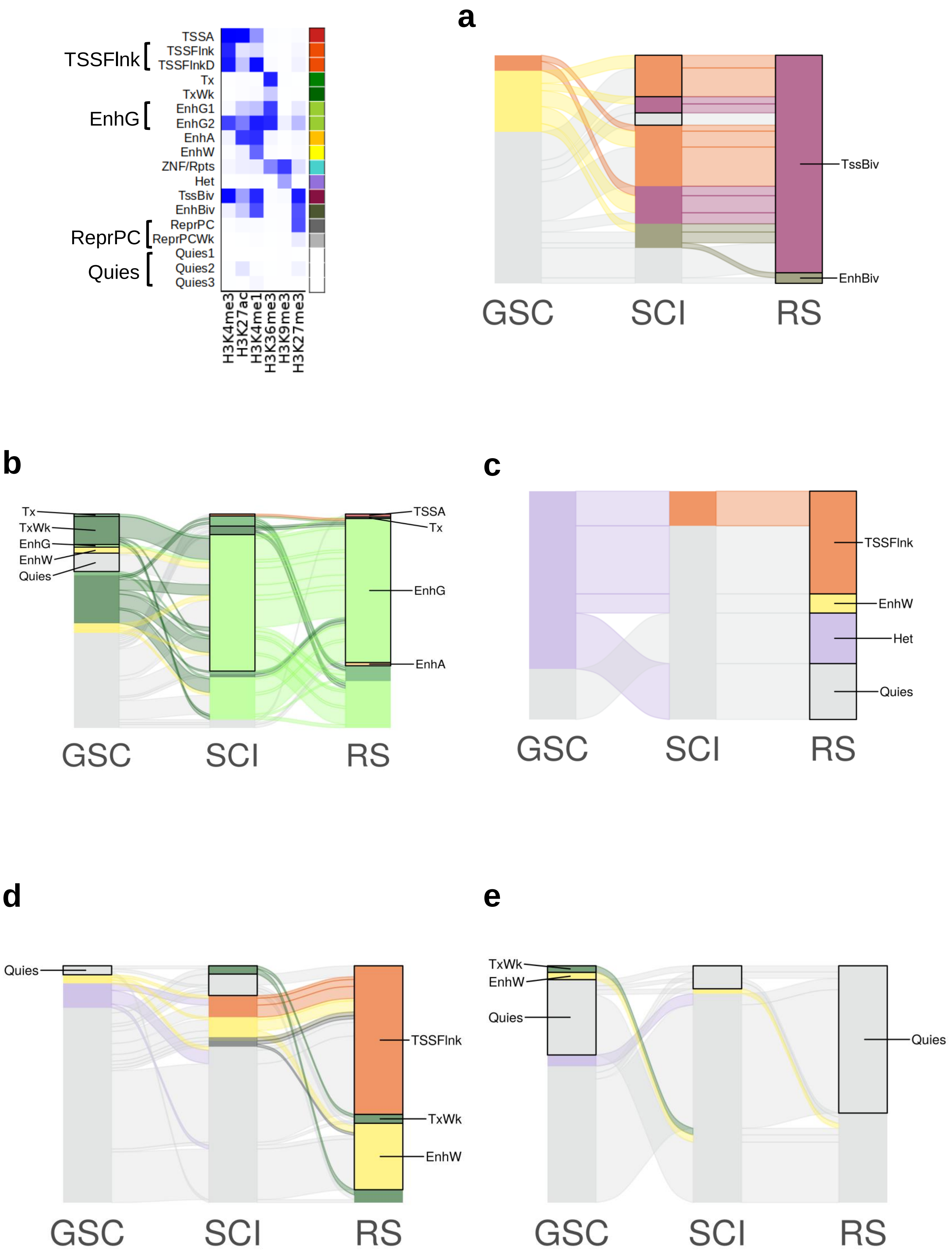

Supplementary Figure 3. Correlation between gene expression and enhancer.

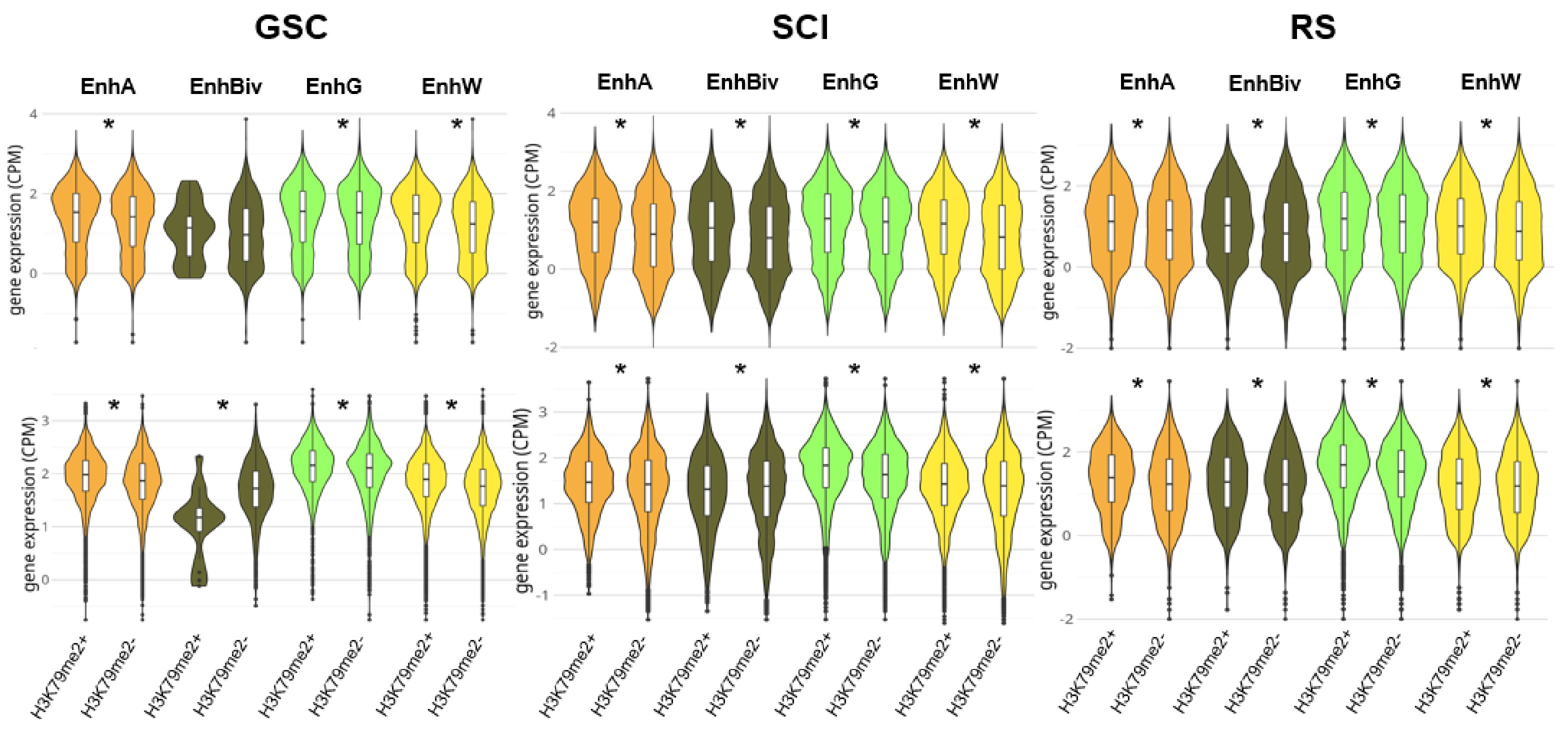

Supplementary Figure 4. *Dot1l-KO*.

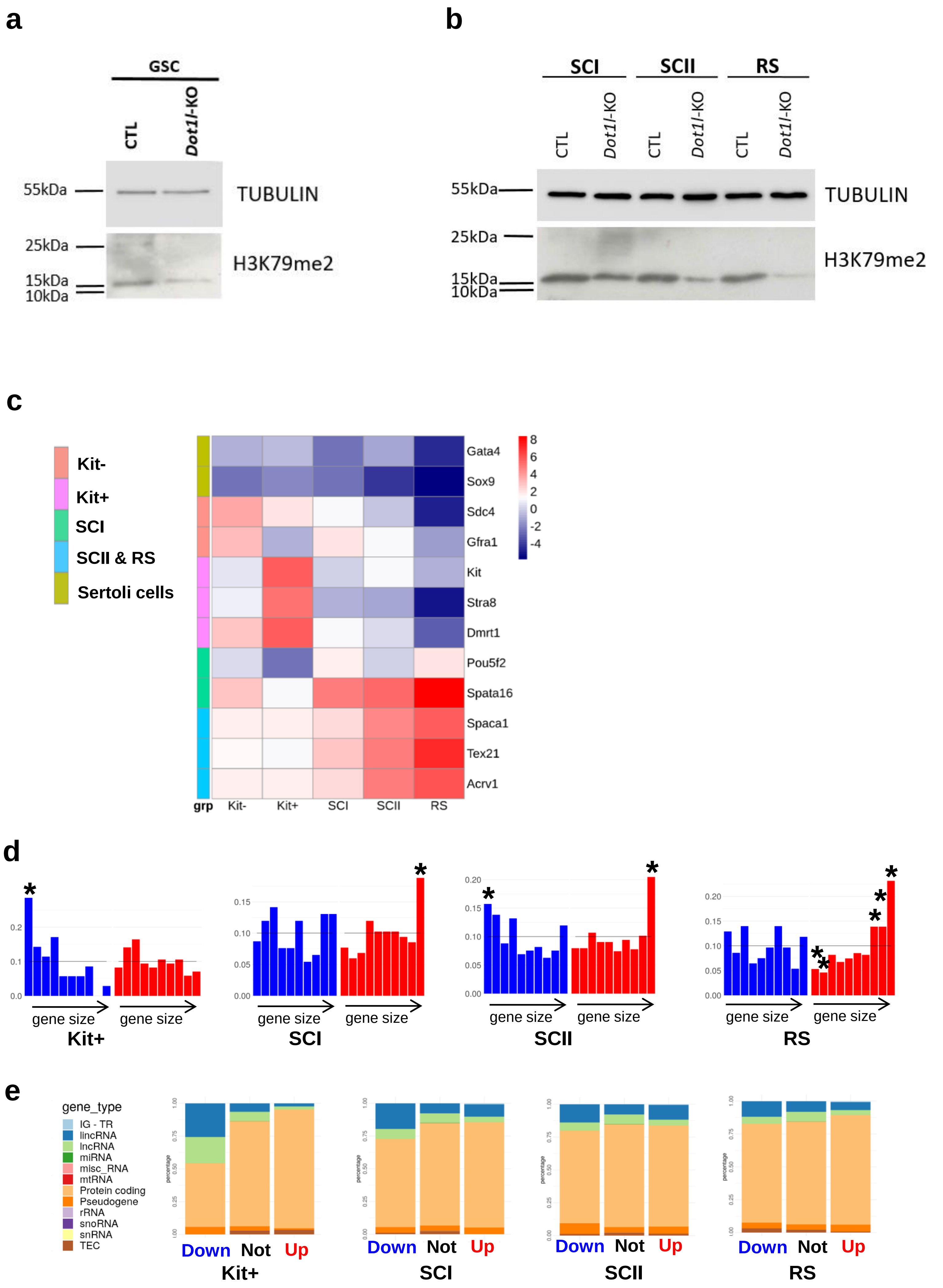

Supplementary Figure 5. *RNA-Seq.*

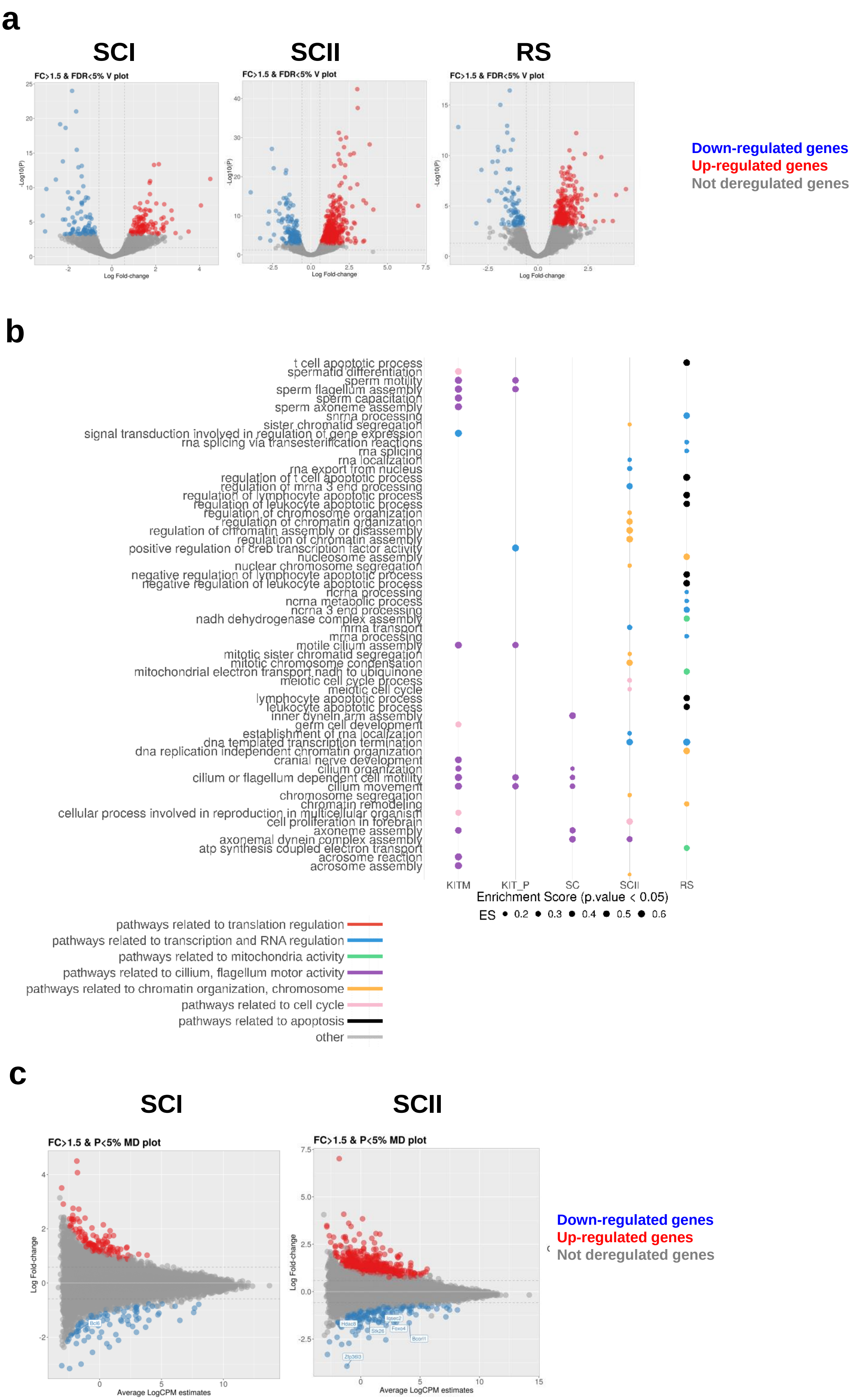

Supplementary Figure 6. Sex chromosome gene regulation.

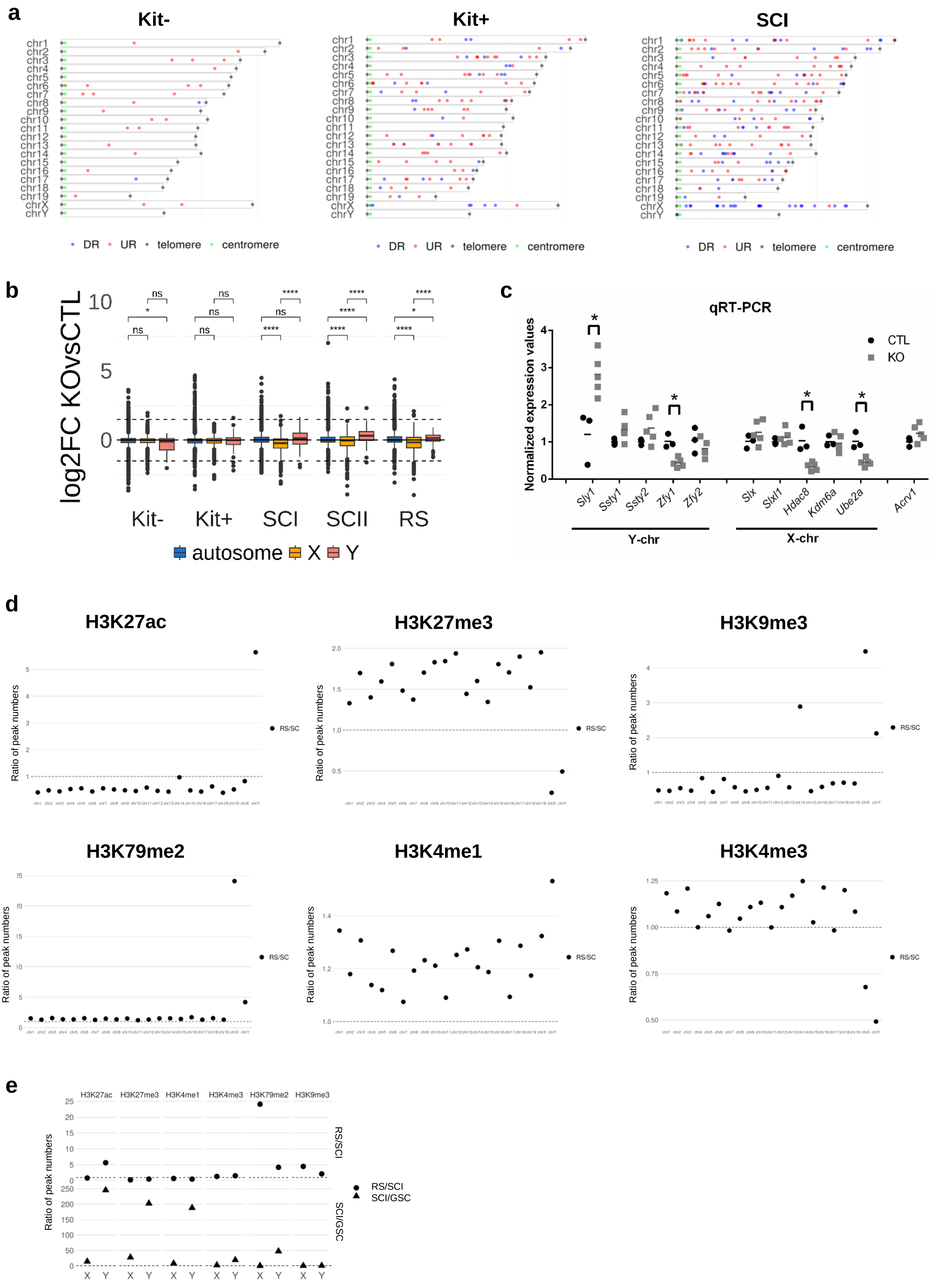

Supplementary Figure 7. ChIP-seq analyses.

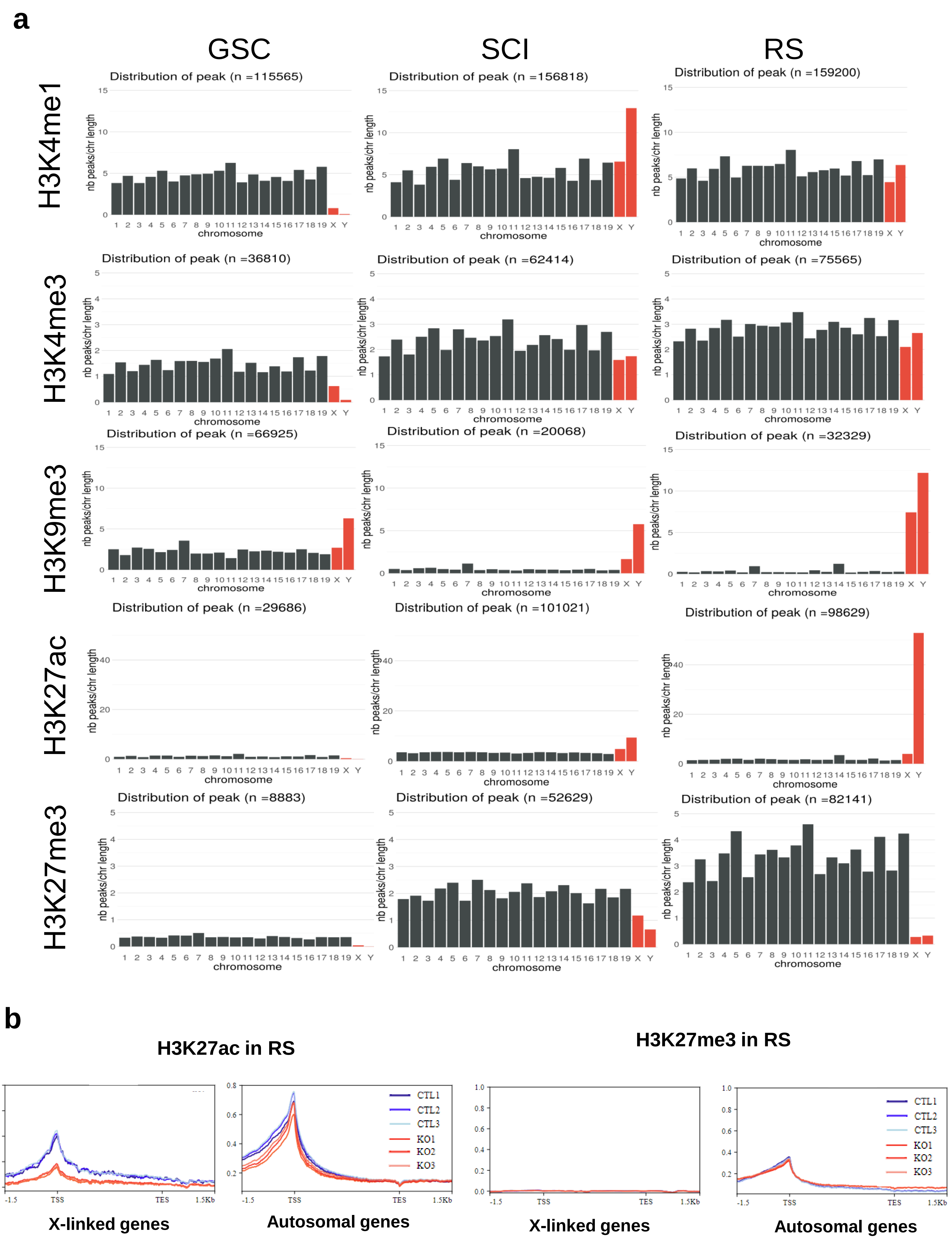

Supplementary Figure 8.

a

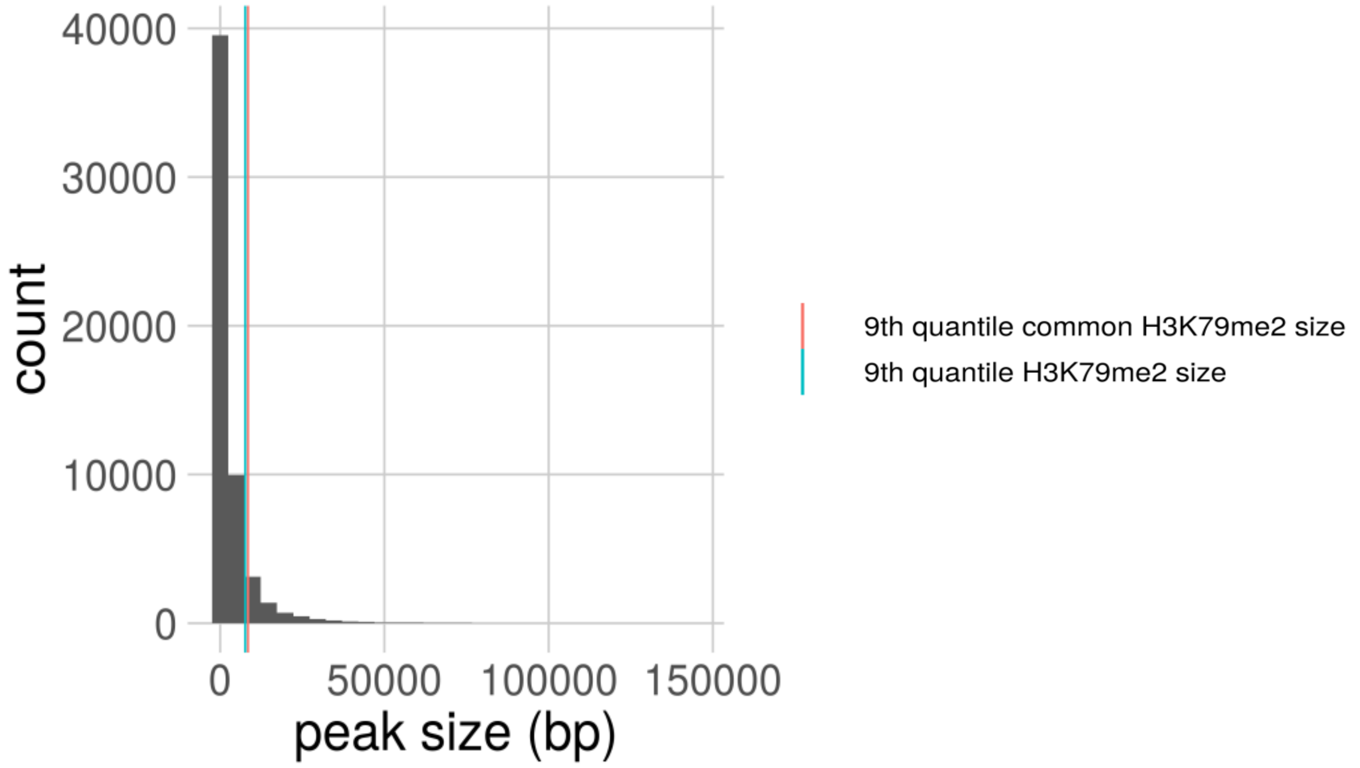

b

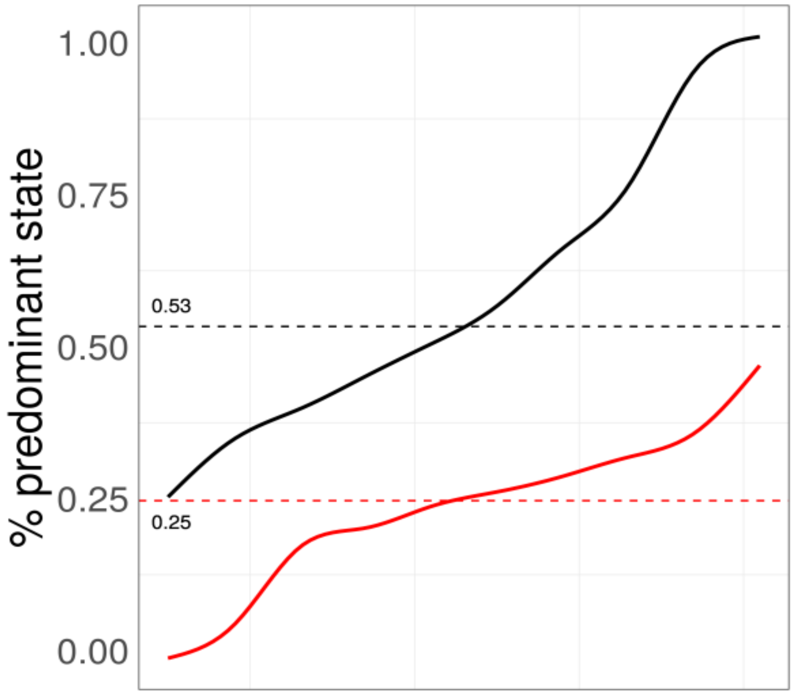

Supplement: Supplementary file 2 — Supplemental material file [file 42003_2024_7393_MOESM2_ESM.pdf]
