# Supplementary material for: Chromatin environment-dependent effects of DOT1L on gene expression in male germ cells
Source: Commun Biol. 2025 Jan 28;8:138. doi: 10.1038/s42003-024-07393-x (PMC11775102; doi:10.1038/s42003-024-07393-x)
Supplement: Supplementary file 3 — Description of Additional Supplementary Files [file 42003_2024_7393_MOESM3_ESM.pdf]

Description of Additional Supplementary Files

File name: Supplementary Data 1

Description: Dynamic of H3K79me2 peaks from GSC to RS.

File name: Supplementary Data 2

Description: DEG genes using spike-in (ERCC) normalization.

File name: Supplementary Data 3

Description: Correlation between DEG and H3K79me2.

File name: Supplementary Data 4

Description: Correlation between DEG and chromatin environment.
