# Supplementary material for: Chromatin environment-dependent effects of DOT1L on gene expression in male germ cells
Source: Commun Biol. 2025 Jan 28;8:138. doi: 10.1038/s42003-024-07393-x (PMC11775102; doi:10.1038/s42003-024-07393-x)
Supplement: Supplementary file 10 — Source data [file 42003_2024_7393_MOESM10_ESM.pdf]

Uncropped Western blot images corresponding to Supplementary Figure 4a

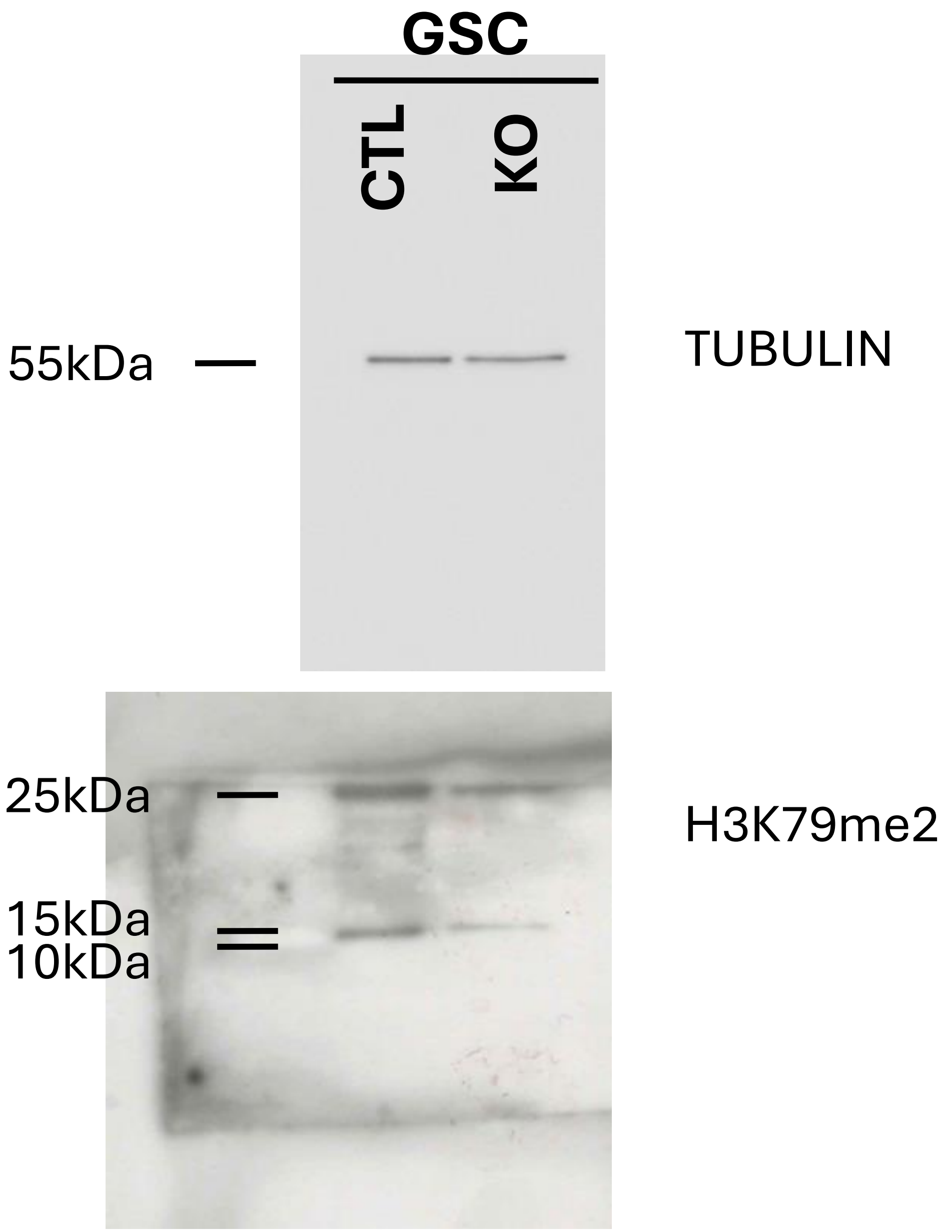

Uncropped Western blot images corresponding to Supplementary Figure 4b

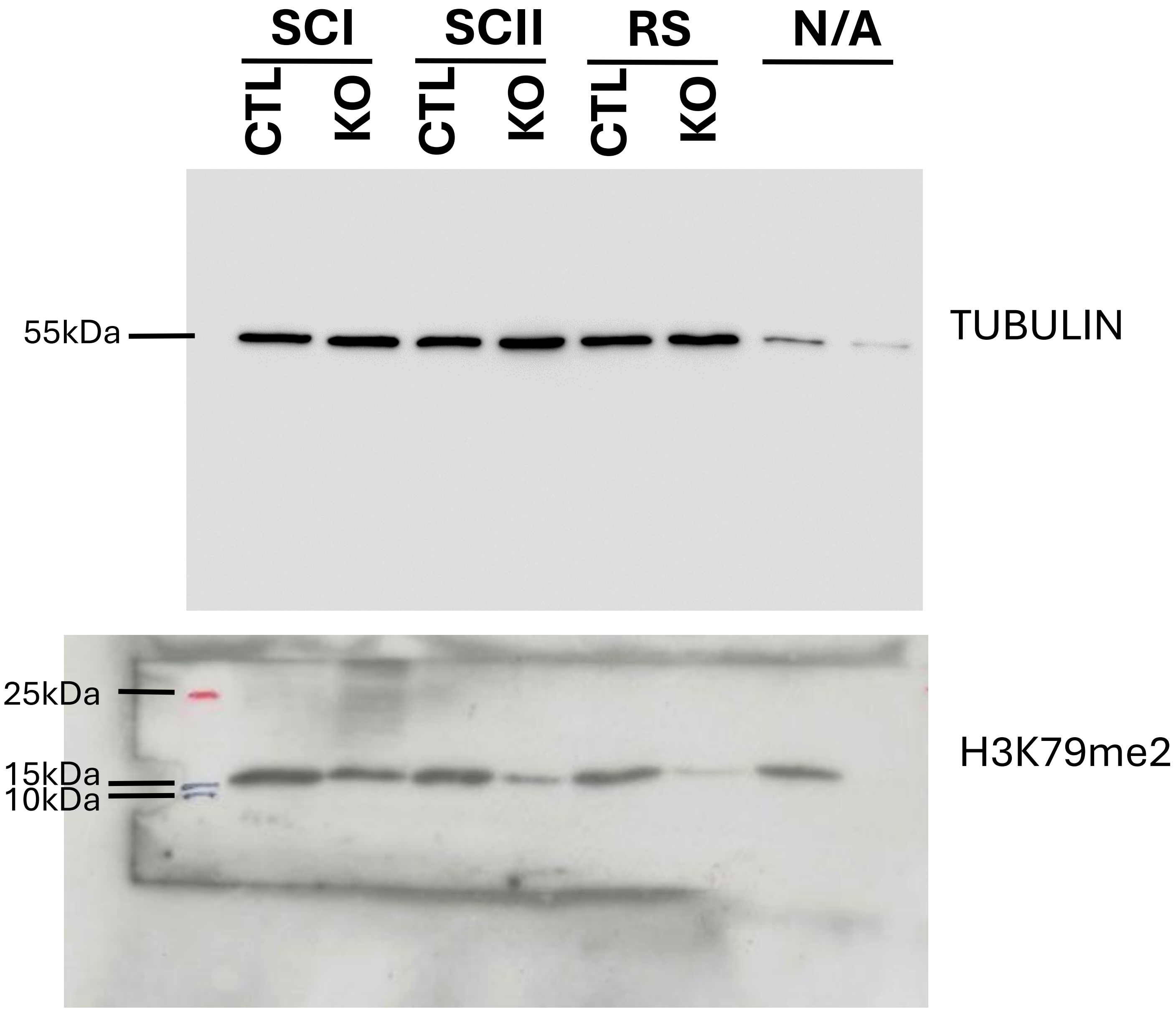

*N/A = samples that are visible in the uncropped images but are not mentioned in the present study
